# Nanoparticle Albumin‐Bound Paclitaxel and Nivolumab for PD‐1 Inhibitor‐Refractory Recurrent or Metastatic Head and Neck Squamous‐Cell Carcinoma

**DOI:** 10.1002/cam4.71533

**Published:** 2026-01-11

**Authors:** Douglas Adkins, Jessica C. Ley, Christine Auberle, Brendan Knapp, Jesse Zaretsky, Jingxia Liu, Peter Oppelt

**Affiliations:** ^1^ Alvin J. Siteman Cancer Center Washington University School of Medicine St. Louis Missouri USA; ^2^ Division of Medical Oncology Washington University School of Medicine St. Louis Missouri USA; ^3^ Robert Ebert and Greg Stubblefield Head and Neck Tumor Center Washington University School of Medicine St. Louis Missouri USA; ^4^ Division of Public Health Sciences, Department of Surgery Washington University School of Medicine St. Louis Missouri USA; ^5^ Division of Biostatistics Washington University School of Medicine St Louis Missouri USA

**Keywords:** head and neck cancer, *nab*‐paclitaxel, nivolumab, recurrent and metastatic disease

## Abstract

**Introduction:**

Standard therapy for PD‐1 inhibitor‐refractory recurrent or metastatic head and neck squamous‐cell carcinoma (RM‐HNSCC) has limited activity. Drugs bound to albumin selectively target cancer cells with upregulated macropinocytosis, a process driven by constitutively hyper‐activated EGFR/RAS/PIK3CA signaling, which is common in HNSCC. Nanoparticle albumin‐bound (*nab*)‐paclitaxel is active in PD‐1 inhibitor‐naïve RM‐HNSCC and alters immune cells to potentially prime tumor response and reverse resistance to PD‐1 inhibitors.

**Methods:**

In a single‐arm phase 2 trial, patients with PD‐1 inhibitor‐refractory RM‐HNSCC received *nab‐*paclitaxel and nivolumab given in 28‐day cycles. The primary endpoint was objective response rate (ORR), using RECIST1.1. Key secondary endpoints were duration of response (DoR), progression‐free survival (PFS), and overall survival (OS). At least one tumor response assessment was required to be evaluable for ORR and PFS. A Simon optimal two‐stage design tested the primary hypothesis (ORR: H_1_ ≥ 50 vs. H_0_ ≤ 30%, type I error 0.05; power 0.80). H_0_ was rejected if ≥ 19 responses were observed in 46 patients. This sample size had a power of 80% to detect the difference in the key secondary hypothesis (median PFS: H_1_ 6.0 vs. H_0_ 3.6 months, two‐sided, one‐sample log rank test; type I error 0.05).

**Results:**

From 9/28/2021–1/4/2024, 46 patients enrolled into the trial. One patient was not evaluable for ORR and PFS. Tumor response occurred in 21 of 45 evaluable patients (ORR 46.7%, 95% CI: 33.8–59.9; vs. H_0_, *p* = 0.0073) and included confirmed response in 20 and unconfirmed response in 1. The best tumor response was complete (4), partial (17), stable (18), and progression (6). The median DoR was 6.1 months (95% CI: 2.8–9.4). With a median follow‐up of 14.1 months (IQR: 7.6–20.1), the median PFS was 5.5 months (95% CI: 3.9–7.8) and the median OS was 13.9 months (95% CI: 9.0–18.9). Treatment‐related deaths did not occur.

**Conclusion:**

Among patients with PD‐1 inhibitor‐refractory RM‐HNSCC, *nab*‐paclitaxel and nivolumab resulted in an ORR and median PFS that were better than historically reported with standard therapy.

**Trial Registration:**

Trial registered on www.clinicaltrials.gov, National Clinical Trial (NCT) 04831320

## 1 Introduction

Over one million new cases of head and neck squamous‐cell carcinoma (HNSCC) occur each year. One‐half of these patients will develop recurrent or metastatic (RM) disease after curatively intended therapy [1, 2]. The current first‐line therapy for patients with RM‐HNSCC includes a PD‐1 inhibitor, either pembrolizumab or nivolumab [3, 4]. However, most patients do not experience benefit with this therapy. The efficacy of subsequent‐line therapy is modest. The best characterized data are with cetuximab, which resulted in an objective response rate (ORR) of 23.9%, a median progression‐free survival (PFS) of 3.8 months, and a median overall survival (OS) of 8.6 months [5]. Similar outcomes were reported by others [6]. Novel strategies are needed to improve the efficacy of therapy for PD‐1 inhibitor‐refractory RM‐HNSCC.

Drugs bound to albumin selectively target cancer cells with upregulated macropinocytosis, an endocytic nutrient‐scavenging process driven by constitutive hyper‐activation of the EGFR/RAS/PIK3CA signaling and Syndecan1 pathways [7, 8, 9, 10, 11, 12]. These pathways are frequently activated in HNSCC and occur with similar frequency across human papillomavirus (HPV) positive and negative subtypes (61% and 62%, respectively) and distant and local‐regional sites of metastases [13, 14, 15]. Nanoparticle albumin‐bound (*nab*)‐paclitaxel is an albumin‐bound formulation of paclitaxel. In preclinical cancer models, intracellular drug concentrations and antitumor activity were higher with *nab*‐paclitaxel than with paclitaxel [16, 17]. In clinical trials across several cancer types, response rates were higher with *nab*‐paclitaxel compared to paclitaxel [18, 19, 20, 21]. Prior to the introduction of PD‐1 inhibitors into clinical practice, we and others reported that *nab*‐paclitaxel is an active agent in locally advanced, untreated RM, and taxane‐resistant HNSCC [22, 23, 24, 25, 26, 27, 28, 29, 30, 31, 32, 33].

Preclinical studies showed that *nab*‐paclitaxel and other chemotherapy agents induced changes in immune and tumor cells that could potentially prime response and reverse resistance to PD‐1 inhibitors. These changes included decreased regulatory T cells, increased pro‐inflammatory macrophages and differentiation of myeloid‐derived suppressor cells to dendritic cells, increased HLA class I expression and antigen presentation, recruitment of CD8+ cytotoxic T cells into tumor, and tumor neoantigen expression [34, 35, 36]. These changes may explain the enhanced tumor response and time to progression or death on second line therapy (PFS2) with chemotherapy, particularly with taxane agents, given for PD‐1 inhibitor‐refractory RM‐HNSCC [37, 38, 39, 40, 41]. Furthermore, concentrations of PD‐1 inhibitors persist for months after their discontinuation due to their long half‐life, and this may also enhance the efficacy of subsequent chemotherapy.

Collectively, these data pointed to *nab*‐paclitaxel as a rational agent to investigate for treatment of PD‐1 inhibitor‐refractory RM‐HNSCC. Although continuation of nivolumab alone after disease progression has limited efficacy, we choose to combine *nab*‐paclitaxel with nivolumab based on the potential for *nab*‐paclitaxel to prime response and reverse resistance to PD‐1 inhibitors [42]. The key aims of this single‐arm phase 2 trial were to determine the ORR and PFS with *nab*‐paclitaxel and nivolumab given to treat patients with PD‐1 inhibitor‐refractory RM‐HNSCC. Herein we report the results of this trial.

## 2 Methods

### 2.1 Study Design and Participants

We performed a single‐center, single‐arm, phase 2 trial, approved by the Washington University (St. Louis, MO, USA) Human Research Protection Office. All study participants provided written, signed, informed consent to participate. The trial was conducted in accordance with Good Clinical Practice guidelines. The Washington University Quality Assurance and Scientific Monitoring committee performed independent data monitoring of the trial. The trial used a Simon optimal two‐stage design to assess the ORR with *nab*‐paclitaxel and nivolumab given to treat PD‐1 inhibitor‐refractory RM‐HNSCC. The full protocol is available in the Appendix S1.

Eligible patients were 18 years or older with RM‐HNSCC originating in the oral cavity, oropharynx, larynx, hypopharynx, or p16 positive SCC of the upper neck nodes with unknown primary tumor site. Patients were required to have measurable disease per Response Evaluation Criteria in Solid Tumors version 1.1 (RECIST v1.1) and disease progression on a PD‐1 inhibitor given alone or with other therapy to treat RM disease, as assessed by RECIST [43]. Other inclusion criteria included Eastern Cooperative Oncology Group (ECOG) performance status 0–1 and adequate marrow and organ function (absolute neutrophil count ≥ 1500/mcL; hemoglobin ≥ 9.0 g/dL; platelets ≥ 100,000/mcL; total bilirubin ≤ 1.5 mg/dL; aspartate (AST) and alanine (ALT) aminotransferase ≤ 2.5× upper limits of normal [ULN]; creatinine ≤ 1.5× ULN or creatinine clearance ≥ 50 mL/min/1.73 m^2^).

Key exclusion criteria included grade ≥ 3 peripheral neuropathy, prior taxane (paclitaxel or docetaxel) given to treat RM disease, corticosteroid in a dose exceeding 10 mg/day of prednisone equivalent or other immunosuppressive therapy, and immunodeficiency disease. A full list of inclusion and exclusion criteria is shown in the protocol.

### 2.2 Procedures

Before treatment, patients underwent history taking, physical examination, complete blood count (CBC), metabolic and thyroid panels, and computerized tomography (CT) scans. Patients were scheduled to receive 28‐day cycles of *nab*‐paclitaxel (125 mg/m^2^ intravenously [IV] on days 1, 8, and 15) and nivolumab (480 mg IV day 1). Criteria for dose reductions, delays, or discontinuation of each study drug are shown in the protocol. Two dose reduction levels of *nab*‐paclitaxel were permitted (100 mg/m^2^ and 75 mg/m^2^). In general, *nab*‐paclitaxel‐related grade 3 or 4 AEs were managed with dose reductions, delays, or discontinuation. No dose reduction levels of nivolumab were allowed. If a study drug was held or discontinued, the other study drug could be given. CT scans were performed after cycle 2, and then after every two cycles. Treatment continued until disease progression, unacceptable toxicity, patient withdrawal, or death. After treatment discontinuation, patients were followed to monitor adverse events (AEs; for at least 100 days), disease status (if appropriate), subsequent lines of anticancer treatment, and survival.

Tumor response was assessed by an independent radiologist using RECIST1.1 [43]. AEs were graded using the National Cancer Institute Common Toxicity Criteria for Adverse Events (NCI‐CTCAE) version 5.0. The investigator judged the relationship of the AEs to the study drugs.

PD‐L1 expression was assessed by immunohistochemistry (IHC) using the DAKO 22C3 clone, and the combined positive score (CPS) was reported. p16 expression was assessed by IHC on tumors of the oropharynx or upper neck nodes with unknown primary tumor site. Tumors with strong (≥ 70%) and diffuse staining for p16 were scored as positive; otherwise, tumors were scored as negative. HPV status was defined as HPV‐positive (p16 positive SCC of the oropharynx or upper neck nodes with unknown primary tumor site) or HPV‐negative (p16 negative SCC of the oropharynx and tumors of all other primary sites).

### 2.3 Outcomes

The primary endpoint was ORR, defined as the proportion of patients with a complete or partial best overall response. Secondary endpoints included PFS (defined as the time from date of first study treatment until date of progression or death, whichever occurred first. Alive patients without progression were censored at date of last follow‐up), the duration of response (DoR: defined as the first date of response until the date of progression or the last date of follow‐up without progression), OS (defined as the time from date of first study treatment to death. Alive patients were censored at date of last follow‐up), the proportion of patients with any grade, grade 1 and 2, and grade 3 or 4 or 5 AEs, and dose delivery (including reductions, delays, and interruptions).

### 2.4 Statistical Analysis

The primary hypothesis of the trial was that *nab*‐paclitaxel and nivolumab would result in a higher ORR than the historical ORR with systemic therapy given to treat PD‐1 inhibitor‐refractory RM‐HNSCC reported in the largest multi‐center study on the date of trial initiation [37]. An ORR of 50% or higher was of clinical interest (alternative hypothesis, H_1_) and an ORR of 30% or lower was deemed unacceptable (null hypothesis, H_0_). Based on Simon's optimal two‐stage design, 15 patients would be enrolled in the first stage, and if 6 or more objective responses were observed, an additional 31 patients would be enrolled in the second stage. Five or fewer objective responses during the first stage would prompt early closure of the trial. Nineteen or more objective responses among the 46 patients accrued during the two stages would be sufficient evidence that this regimen was efficacious and worthy of further investigation. The regimen was not worthy of further investigation if 18 or fewer objective responses occurred in 46 patients. This design would detect the difference of ORRs between H_1_ and H_0_, with an 80% power at a one‐sided 0.05 significance level. The ORR was estimated with 95% CIs and compared to H_0_ using a binomial distribution.

The key secondary hypothesis of the trial was that *nab*‐paclitaxel and nivolumab would result in a longer median PFS than historically reported with systemic therapy given to treat PD‐1 inhibitor‐refractory RM‐HNSCC in the largest muti‐center study at the time of trial initiation [37]. Using a historical median PFS of 3.6 months, a sample size of 46 evaluable patients had at least an 80% power to detect the difference when the median PFS with *nab*‐paclitaxel and nivolumab was 6.0 months using a two‐sided, one‐sample log rank test at the Type I error of 0.05.

DoR was summarized by using median and interquartile range (IQR) values. PFS and OS were estimated using the Kaplan–Meier method. AEs were summarized by type and grade. Drug delivery was summarized by proportion of patients who did and did not experience dose reductions, delays, and interruptions.

Consistent with the historical reference report, patients who received at least one dose of study treatment and underwent at least one tumor response assessment were evaluable for ORR, DoR, and PFS [37]. Patients who received at least one dose of study treatment were evaluable for OS, AEs, and drug delivery.

The statistical analysis was performed using SAS version 9.4 (SAS Institute, Cary, NC) or R version 4.3.3. This study is registered [with ClinicalTrials.gov, number NCT04831320].

### 2.5 Role of the Funding Source

Funding and study drugs were provided by Bristol‐Myers Squibb, Princeton, NJ, USA. Bristol‐Myers Squibb had no role in the study design, data collection, data analysis, data interpretation, or writing of the report. The data analysis and interpretation were performed by the authors. All authors had full access to all data in the study. The corresponding author had final responsibility for the decision to submit for publication.

## 3 Results

**FIGURE 1:**
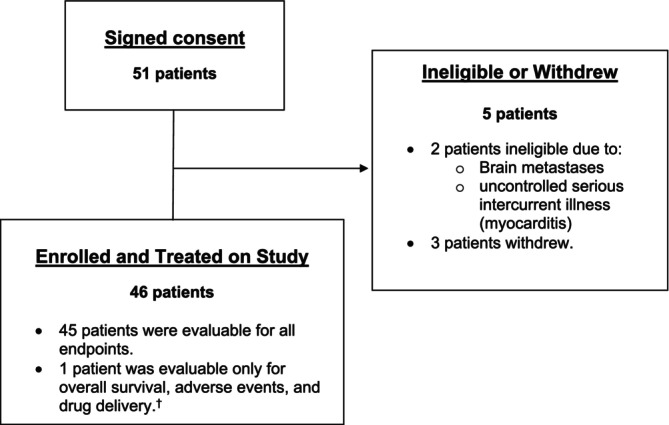
Trial profile. ^†^Patient did not undergo a response assessment while on the trial.

The trial profile is shown in Figure 1. Between 28 September 2021 and 4 January 2024, 46 patients enrolled and were treated with study drugs. Forty‐five patients were evaluable for all endpoints. One patient was not evaluable for ORR, DoR, or PFS but was evaluable for OS, AEs, and drug delivery. The cut off for data analysis presented here was 12 November 2024. Median follow‐up was 14.1 months (IQR 7.6–20.1).

Baseline patient and tumor characteristics for all 46 patients are shown in Table 1. Forty (87.0%) patients were male, 6 (13.0%) were female, 42 (91.3%) were White, and 4 (8.7%) were Black or African American. HPV disease status was positive in 22 patients (47.8%) and negative in 24 patients (52.2%). PD‐L1 CPS disease status was ≥ 1 in 42 patients (91.3%) and 0 in 4 (8.7%) patients. Twelve (26.1%) patients experienced a tumor response with prior PD‐1 inhibitor‐based therapy. Thirty‐eight (82.6%) patients enrolled within 3 months of last dose of prior PD‐1 inhibitor.

**TABLE 1:** Baseline patient and tumor characteristics.

Characteristic	*N* = 46
Age	64.3
Median (IQR) years	60.3–67.4
Sex^a^	
Male	40 (87.0)
Female	6 (13.0)
Race^a^	
White	42 (91.3)
Black or African American	4 (8.7)
Performance status^a^	
0	34 (73.9)
1	12 (26.1)
Smoking history^a^	
Yes	29 (63.0)
No	17 (37.0)
HPV^b^ status and primary site^a^	
HPV positive (oropharynx)	22 (47.8)
HPV negative	24 (52.2)
Oral cavity	14
Oropharynx	4
Larynx	4
Hypopharynx	2
Site of recurrence at study entry^a^	
Local/regional	4 (8.7)
Distant metastasis	20 (43.5)
Both	22 (47.8)
PD‐L1 CPS^a^	
≥ 1	42 (91.3)
≥ 20	20
1–19	22
0	4 (8.7)
# lines of prior systemic therapy for RM^c^ disease^a^	
1	32 (69.6)
2	11 (23.9)
≥ 3	3 (6.5)
Months from prior PD‐1 inhibitor^a^	
≤ 3	38 (82.6)
4–11	4 (8.7)
≥ 12	4 (8.7)
Months on prior PD‐1 inhibitor, median [IQR]	3.4 (2.1–8.5)
≤ 6	34 (73.9)
7–11	5 (10.9)
≥ 12	7 (15.2)
Best response to prior PD‐1 inhibitor^a^	
CR	2 (4.3)
PR	10 (21.7)
SD	15 (32.6)
PD	19 (41.3)
Prior platinum for RM^c^ disease or RM^c^ disease within 6 months of platinum for LA^d^ disease^a^	
Yes	31 (67.4)
No	15 (32.6)

^a^ Number (%) of patients with characteristic.

^b^ Human papillomavirus.

^c^ Recurrent or metastatic.

^d^ Locally advanced.

**FIGURE 2:**
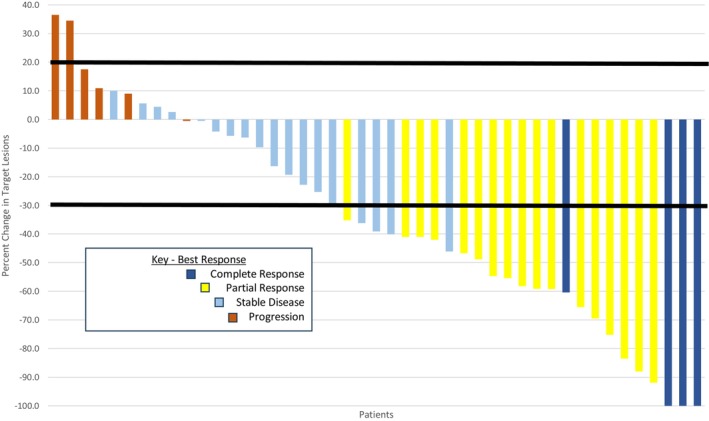
Waterfall Plot showing the best response (percent change) in target lesions for the 45 evaluable patients. *Four patients had progression due to new radiographic lesions (2 patients) or increase of lesions observed on clinical examination (2 patients). Five patients with ≥ 30% decrease in target lesion(s) had stable disease because subsequent imaging did not confirm partial response. One patient had complete response but < 100% decrease in target lesion(s) because the remaining target lesion was a lymph node that measured < 1 cm.

The ORR was 46.7% (95% CI: 33.8–59.9) [versus H_0_ ORR ≤ 30%, one‐sided *p* = 0.0073]. Tumor response occurred in 21 of 45 evaluable patients and included confirmed response in 20 and unconfirmed response in 1. The best tumor response was complete in 4 patients (8.8%), partial in 17 (37.8%), stable in 18 (40%), and progressive in 6 (13.3%). The median DoR was 6.1 months (95% CI: 2.8–9.4). A waterfall plot that displays the best response (percent change) in the target lesions for each of the 45 evaluable patients is shown in Figure 2.

Tumor response occurred numerically more frequently patients with HPV‐positive versus HPV‐negative disease (68.2%: 15 of 22 patients vs. 26.1%: 6 of 23) and in patients with distant metastasis (DM) only versus local‐regional recurrence (LRR) with or without DM (68.4%: 13 of 19 vs. 30.8%: 8 of 26). Tumor response occurred with similar frequency in patients with PD‐L1 CPS ≥ 20 versus CPS 0–19 disease (40%: 8 of 20 vs. 52%: 13 of 25), in patients with versus without tumor response to prior PD‐1 inhibitor (50%: 6 of 12 vs. 45.5%: 15 of 33), and in patients who enrolled ≤ 3 months versus > 3 months since completion of prior PD‐1 inhibitor (47.4%: 18 of 38 vs. 37.5%: 3 of 8).

**FIGURE 3:**
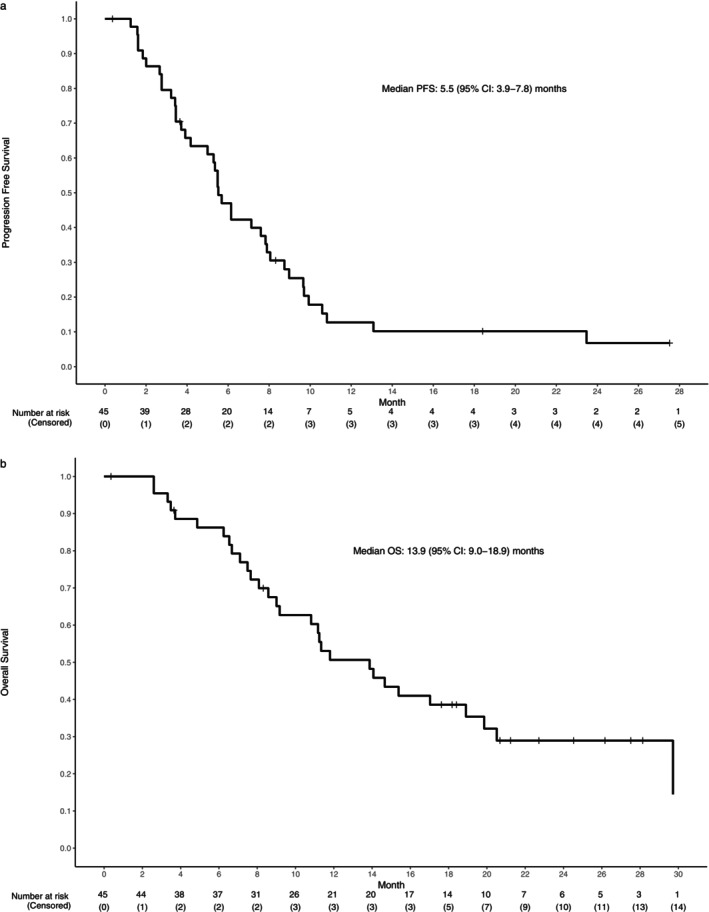
Progression‐free survival (PFS) and overall survival (OS) for the evaluable patients.

Among the 45 evaluable patients, the median PFS was 5.5 months (95% CI: 3.9–7.8). Among all 46 patients, the median OS was 13.9 months (95% CI: 9.0–18.9) (Figure 3). The survival analyses of subgroups based on PD‐L1 CPS and HPV status and interval from prior PD‐1 inhibitor are shown in the Appendix S1 (Figure S1, pp. 1). Among the 44 patients removed from the study, subsequent lines of systemic treatment were administered to 33 (75%) and included one additional line in 19 and two or more lines in 14.

At last follow‐up, 16 of the 46 patients were alive and 30 had died. Of the 16 living patients, 2 had disease control and continued with study therapy and 14 discontinued study therapy (all due to disease progression). Causes of death for all 30 expired patients were disease progression (27) and AEs attributed to subsequent lines of therapy given after disease progression on *nab*‐paclitaxel and nivolumab (3).

AEs that occurred among the 46 patients are shown in Table 2. Grade 3 AEs of any cause occurred in 39 patients (84.7%), grade 4 AEs in 12 (26.1%) and grade 5 AEs in 5 (10.9%). Treatment‐related deaths did not occur. Most of the grade 3 AEs were unrelated to the study drug(s). Ten of the grade 4 AEs (4‐neutropenia, 3‐leukopenia, 1‐anemia, 1‐sepsis, and 1‐respiratory failure) were related to study drug(s). Febrile neutropenia (grade 3) occurred in one patient (2.2%). The most common AEs of any grade and cause were lymphopenia (97.8%), anemia (89.1%), hyponatremia (80.4%), leukopenia (76.1%), fatigue (67.4%), weight loss (67.4%), neutropenia (65.2%), hypoalbuminemia (63.1%), hypertension (60.9%), and peripheral sensory neuropathy (50.0%). Non‐thyroid, immune‐related AEs (irAEs) occurred in 23.9% of patients, most cases were rash.

**TABLE 2:** Summary of adverse events (*N* = 46 patients)^e^.

AE term	Grade 1–2	Grade 3	Grade 4	Grade 5^f^
Alanine aminotransferase increased	15 (33)	3 (7)	0	0
Alkaline phosphatase increased	17 (37)	2 (4)	0	0
Alopecia	6 (13)	0	0	0
Anemia	28 (61)	12 (26)	1^c^ (2)	0
Aspartate aminotransferase increased	12 (26)	2 (4)	0	0
Aspiration	1 (2)	4 (9)	0	1^a^ (2)
Back pain	2 (4)	1 (2)	0	0
Blood bilirubin increased	5 (11)	0	0	0
Blood lactate dehydrogenase increased	11 (24)	0	0	0
Cardiac arrest	0	0	1^a^ (2)	0
Chronic kidney disease	7 (15)	0	0	0
Constipation	9 (20)	0	0	0
Cough	6 (13)	0	0	0
Creatinine increased	5 (11)	0	0	0
Death NOS	0	0	0	1^a^ (2)
Diarrhea	10 (22)	3 (7)	0	0
Dysphagia	1 (2)	2 (4)	0	0
Dyspnea	6 (13)	0	0	0
Edema face	2 (4)	1 (2)	0	0
Edema limbs	6 (13)	0	0	0
Enterocolitis infectious	1 (2)	1 (2)	0	0
Enterovesical fistula	0	1 (2)	0	0
Eosinophilia	10 (22)	0	0	0
Fall	5 (11)	2 (4)	0	0
Fatigue	24 (52)	7 (15)	0	0
Febrile neutropenia	0	1 (2)	0	0
Fever	5 (11)	0	0	0
Flank pain	0	1 (2)	0	0
Gastroesophageal reflux disease	0	1 (2)	0	0
Gastrointestinal disorders—other, perforated diverticulitis	0	1 (2)	0	0
GGT increased	2 (4)	2 (4)	0	0
Headache	3 (7)	1 (2)	0	0
Hematuria	5 (11)	0	0	0
Hypercalcemia	10 (22)	0	2^a^ (4)	0
Hyperglycemia	12 (26)	1 (2)	0	0
Hyperkalemia	10 (22)	1 (2)	0	0
Hypernatremia	5 (11)	0	0	0
Hypertension	20 (43)	8 (17)	0	0
Hypoalbuminemia	25 (54)	4 (9)	0	0
Hypocalcemia	14 (30)	4 (9)	0	0
Hypokalemia	13 (28)	0	1^a^ (2)	0
Hypomagnesemia	6 (13)	0	0	0
Hyponatremia	35 (76)	2 (4)	0	0
Hypophosphatemia	13 (28)	0	0	0
Hypotension	6 (13)	1 (2)	0	0
Infusion related reaction	1 (2)	0	0	1^a^ (2)
Localized edema	1 (2)	2 (4)	0	0
Lung infection	1 (2)	6 (13)	1^a^ (2)	0
Lymphocyte count decreased	20 (43)	17 (37)	8^a^ (17)	0
Meningitis	0	1 (2)	0	0
Mucositis oral	9 (20)	3 (7)	0	0
Muscle weakness lower limb	0	2 (4)	0	0
Myalgia	2 (4)	1 (2)	0	0
Nausea	14 (30)	1 (2)	0	0
Neck pain	2 (4)	0	0	0
Neutrophil count decreased	17 (37)	8 (17)	5^a(1), c(4)^ (11)	0
Oral hemorrhage	0	1 (2)	1^b^ (2)	0
Oral pain	1 (2)	1 (2)	0	0
Pericardial effusion	0	1 (2)	0	0
Peripheral motor neuropathy	2 (4)	1 (2)	0	0
Peripheral sensory neuropathy	21 (46)	2 (4)	0	0
Platelet count decreased	20 (43)	2 (4)	0	0
Pleural effusion	1 (2)	2 (4)	0	0
Pneumothorax	0	1 (2)	0	0
Proteinuria	7 (15)	0	0	0
Pulmonary edema	0	1 (2)	0	0
Rash maculo‐papular	12 (26)	2 (4)	0	0
Respiratory failure	0	0	1^d^ (2)	0
Salivary gland infection	0	1 (2)	0	0
Sepsis	0	1 (2)	2^a(1), c(1)^ (4)	1^a^ (2)
Skin infection	0	2 (4)	0	0
Soft tissue infection	0	1 (2)	0	0
Syncope	0	1 (2)	0	0
Thyroid stimulating hormone increased	8 (17)	0	0	0
Tracheal hemorrhage	0	0	0	1^a^ (2)
Tumor pain	4 (9)	1 (2)	0	0
Urinary tract infection	2 (4)	1 (2)	0	0
Vascular access complication	0	1 (2)	0	0
Vomiting	9 (20)	1 (2)	0	0
Weight gain	8 (17)	0	0	0
Weight loss	27 (59)	4 (9)	0	0
White blood cell decreased	20 (43)	10 (22)	5^a(1), b(1), c(3)^ (11)	0

*Note:* Attribution: ^a^Unrelated. ^b^Unlikely related. ^c^Possibly related. ^d^Probably related. In cases of plural attributions, the adjacent number(s) in superscript indicate(s) the number of patients with that AE attribution.

^e^ Grade 1–2 adverse events (AEs) with an incidence of ≥ 10% are shown. All grade 3–5 events shown.

^f^ Grade 5 AEs included aspiration pneumonia (1) and tracheal hemorrhage (1) each attributed to RM‐HNSCC, and sepsis (1) attributed to carboplatin, infusion‐related reaction (1) attributed to cetuximab, and death NOS (1) that occurred on cetuximab and palbociclib each given as subsequent line of therapy after disease progression on *nab*‐paclitaxel and nivolumab.

The number of all patients who required dose modification or hold of each study drug by cycle of treatment is shown in the Appendix S1 (Table S1, pp. 4). The median number of cycles of study drugs administered was 5 (IQR: 2–8). Across all cycles of therapy, 20 patients (43.5%) received *nab*‐paclitaxel and nivolumab without dose modification or hold, whereas 26 (56.5%) required dose modification or hold of either *nab*‐paclitaxel (22) or nivolumab (4). The most common reasons for dose modification or hold of *nab*‐paclitaxel were peripheral sensory neuropathy (8) and neutropenia (7).

## 4 Discussion

In this trial of patients with PD‐1 inhibitor‐refractory RM‐HNSCC, the ORR with *nab*‐paclitaxel and nivolumab was 46.7% (95% CI: 33.8–59.9), which was higher than historically reported with standard therapy (H_0_ ORR ≤ 30%, one‐sided *p* = 0.0073) [37]. The historical ORR referenced here was from the largest multi‐center study reported when our trial began [37]. Previous studies that assessed the efficacy of systemic therapy given for PD‐1 inhibitor‐refractory RM‐HNSCC reported ORRs of 27%–42% [37, 38, 39, 40, 41]. However, these studies were limited by their retrospective design and heterogeneous patient characteristics and systemic therapies. Recently, a phase 3 trial of patients with PD‐1 inhibitor‐refractory RM‐HNSCC showed that cetuximab resulted in an ORR of 23.9%, lower than the historical ORR used to design our trial [5].

In contrast to prior reports of systemic therapy in this patient population, we administered a nanoparticle albumin‐bound formulation of paclitaxel in combination with a PD‐1 inhibitor. Compared to paclitaxel, *nab*‐paclitaxel may enhance antitumor activity by selective targeting of HNSCC cells with upregulated macropinocytosis due to constitutive hyper‐activation of the EGFR/RAS/PIK3CA signaling pathways [7, 8, 9, 10, 11, 12, 13, 14, 15, 16, 17]. Furthermore, *nab*‐paclitaxel‐induced changes in immune and tumor cells could potentially prime response and reverse resistance to PD‐1 inhibitors [34, 35, 36]. Under these conditions, continuation of PD‐1 inhibitor with *nab*‐paclitaxel may further enhance tumor response.

Prior reports of patients with PD‐1 inhibitor‐refractory RM‐HNSCC observed that ORRs tended to be higher in patients who were treated with platinum‐containing regimens or who had experienced tumor response to prior PD‐1 inhibitor [37, 38, 39, 40, 41].

In contrast, the ORRs with *nab*‐paclitaxel and nivolumab were similar in patients with and without tumor response to prior PD‐1 inhibitor but were higher in patients with HPV‐positive versus HPV‐negative disease (68.2 vs. 26.1%) and in patients with only DM versus local‐regional recurrence (LRR) with or without DM (68.4 vs. 30.8%).

Among patients with PD‐1 inhibitor‐refractory RM‐HNSCC, *nab*‐paclitaxel and nivolumab resulted in a median PFS and OS that were better than historically reported with standard therapy. The median PFS was 5.5 months (95% CI: 3.9–7.8) and the median OS was 13.9 months (95% CI: 9.0–18.9). The lower boundary of the 95% CI for the median PFS with *nab*‐paclitaxel and nivolumab was higher than the median PFS of 3.6 months historically reported with standard therapy [37]. Previous retrospective studies reported median PFS of 3.3–4.2 months and median OS of 7.8–9.8 months [37, 38, 39, 40, 41]. A phase 3 trial showed that cetuximab resulted in a median PFS of 3.8 months and a median OS of 8.6 months [5].

In this trial, primary resistance to *nab*‐paclitaxel and nivolumab occurred in 13.3% of patients. The median DoR was 6.1 months (95% CI: 2.8–9.4) and, at last follow‐up, 95.7% of patients had experienced disease progression. These data imply that primary and secondary resistance to systemic therapy remains a significant challenge in patients with PD‐1 inhibitor‐refractory RM‐HNSCC.

*Nab*‐paclitaxel and nivolumab were safe to administer to patients with PD‐1 inhibitor‐refractory RM‐HNSCC. Treatment‐related deaths did not occur. Grade 3 AEs of any cause were frequent, although most were not related to the study drug(s). However, treatment‐related hematologic AEs and peripheral sensory neuropathy were common. Ten patients (21.7%) experienced treatment‐related grade 4 AEs (usually neutropenia and leukopenia, although febrile neutropenia occurred in only one patient). Most patients (56.5%) required dose modification or hold of study drug(s), more frequently *nab*‐paclitaxel, and usually for neutropenia and peripheral neuropathy. Careful monitoring for these AEs along with appropriate dose modifications or holds are key to optimizing dose delivery of this regimen.

Several limitations of this trial exist. This was a single‐center, single‐arm trial with a small sample size. The activity results had wide CIs. Patient and tumor characteristics may have differed from the prespecified historical control group. The study was not designed to determine the independent contribution of nivolumab to the overall activity of the regimen. A concurrent control group treated with an alternative systemic therapy was not part of the study design. The favorable OS observed in our trial may have been influenced by the high proportion of patients (75%) treated with subsequent lines of systemic treatment upon removal from the trial.

Among patients with PD‐1 inhibitor‐refractory RM‐HNSCC, we conclude that *nab*‐paclitaxel and nivolumab resulted in a higher ORR and longer median PFS than historically reported with alternative systemic therapy [37, 38, 39, 40, 41]. These prospective data also provide important benchmarks that can be used to design future studies that test the activity of novel systemic therapy in patients with PD‐1 inhibitor‐refractory RM‐HNSCC.

## Author Contributions

**Douglas Adkins:** conceptualization, data curation, formal analysis, funding acquisition, investigation, methodology, patient enrollment, resources, supervision, validation, visualization, writing – original draft, writing – review and editing. **Jessica C. Ley:** data curation, project administration, resources, supervision, visualization, writing – review and editing. **Christine Auberle:** writing – review and editing. **Brendan Knapp:** writing – review and editing. **Jesse Zaretsky:** writing – review and editing. **Jingxia Liu:** formal analysis, writing – review and editing. **Peter Oppelt:** investigation, patient enrollment, resources, validation, visualization, writing – review and editing.

## Funding

Bristol‐Myers Squibb and generous support from Gregory Stubblefield and Nancy Apel.

## Ethics Statement

The Washington University School of Medicine Institutional Review Board approved the study and the study conforms to the US Federal Policy for the Protection of Human Subjects.

## Conflicts of Interest

J.L. and J.Z. have no disclosures to report. D.A. reports research support (study drugs) from Bristol‐Myers Squibb for the submitted work and consulting or scientific advisory board support from Adlai Nortye, Boehringer Ingelheim, Coherus, Cue Biopharma, Exelixis, Genmab, Immunitas, Inhibrix, Sanofi, Kura Oncology, Merck, Merck KGaA, Merus, Natco Pharma, Purple Biotech, Regeneron, Seagen, and TargImmune Therapeutics; travel support from Natco Pharma, leadership role on the NCCN practice guidelines and the Barnes Jewish Hospital Pharmacy and Therapeutics committee; and institutional research support from Pfizer, Eli Lilly, Merck, Celgene/BMS, Novartis, AstraZeneca, Blueprint Medicine, Kura Oncology, Cue Biopharma, Cofactor Genomics, Debiopharm International, ISA Therapeutics, Gilead Sciences, BeiGene, Roche, Vaccinex, Hookipa Biotech, Adlai Nortye USA, Epizyme, BioAtla, Boehringer Ingelheim, Calliditas Therapeutics, Genmab, Natco Pharma, Tizona Therapeutics, Erasca, Alentis, Inhibrx, Seagen, Coherus, Takeda, Xilio, GSK, Johnson & Johnson, Immunotep, Exelixis, Daiichi Sankyo, Janux, Merus, Tempus, Aveo Pharmaceuticals, and Rgenta outside the submitted work. J.C.L. reports travel support from Natco Pharma, outside the submitted work. C.A. reports honoraria from PrecisCa and OncLive, outside the submitted work. B.K. reports honoraria from Targeted Oncology, outside the submitted work. P.O. reports research funding from Merck and HiFiBio and travel support from Natco Pharma, outside the submitted work.

## Supporting information


**Appendix S1:** cam471533‐sup‐0001‐AppendixS1.docx.

## Data Availability

Individual participant data that underlie the results reported in this article, after de‐identification (text, tables, figures, and appendices), and the study protocol will be shared, beginning 9 months and ending 24 months following article publication, with investigators whose proposed use of the data has been approved by an independent review committee (“learned intermediary”) identified for this purpose. Types of acceptable analyses include approved proposal(s) or individual participant data for meta‐analyses. Proposals may be submitted up to 24 months following article publication. Information regarding submitting proposals and accessing data may be submitted to jcley@wustl.edu.
