# Supplementary material for: Nanoparticle Albumin‐Bound Paclitaxel and Nivolumab for PD‐1 Inhibitor‐Refractory Recurrent or Metastatic Head and Neck Squamous‐Cell Carcinoma
Source: Cancer Med. 2026 Jan 11;15(1):e71533. doi: 10.1002/cam4.71533 (PMC12791152; doi:10.1002/cam4.71533)

**Table S1:** Number of patients who required dose modification or hold of each study drug by treatment cycle interval.

| **Characteristic** | **Cycle Number** | | |
| --- | --- | --- | --- |
|  | **1-2** | **3-6** | **7+** |
| **Evaluable Patients (n)** | 46 | 34 | 16 |
| ***nab*-paclitaxel (n)** | 8 | 11 | 11 |
| **Nivolumab (n)** | 0 | 3 | 1 |

**Appendix Figure Legend**

Survival analyses (PFS and OS) of subgroups based on:

a. PD-L1 CPS (0-19 or 20-100)

b. HPV status (negative or positive)

c. Interval from prior PD-1 inhibitor (less than or more than 6 months)


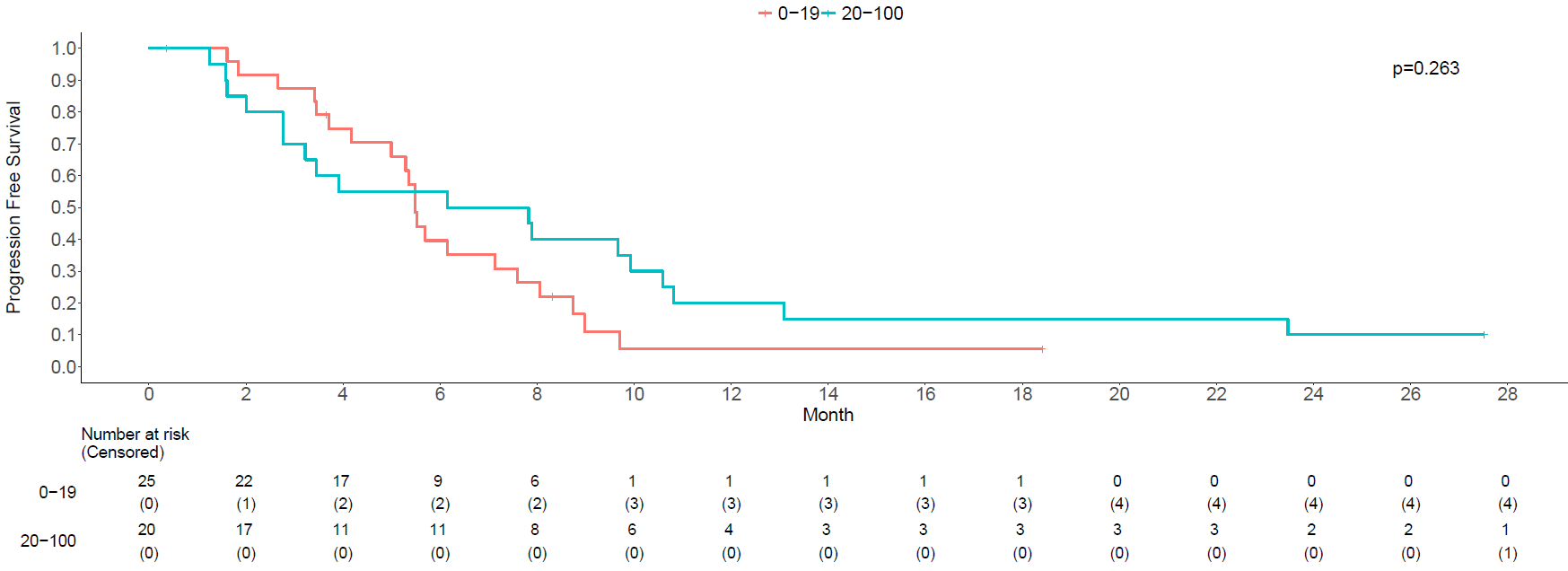


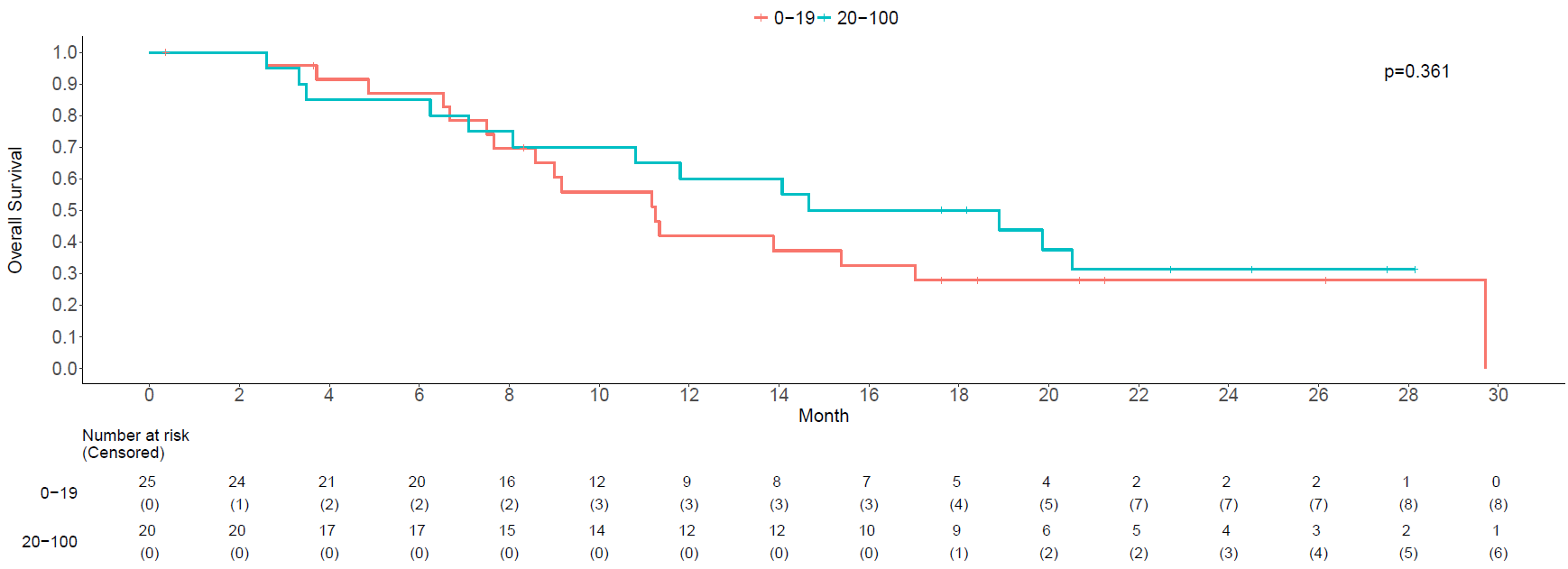


b.
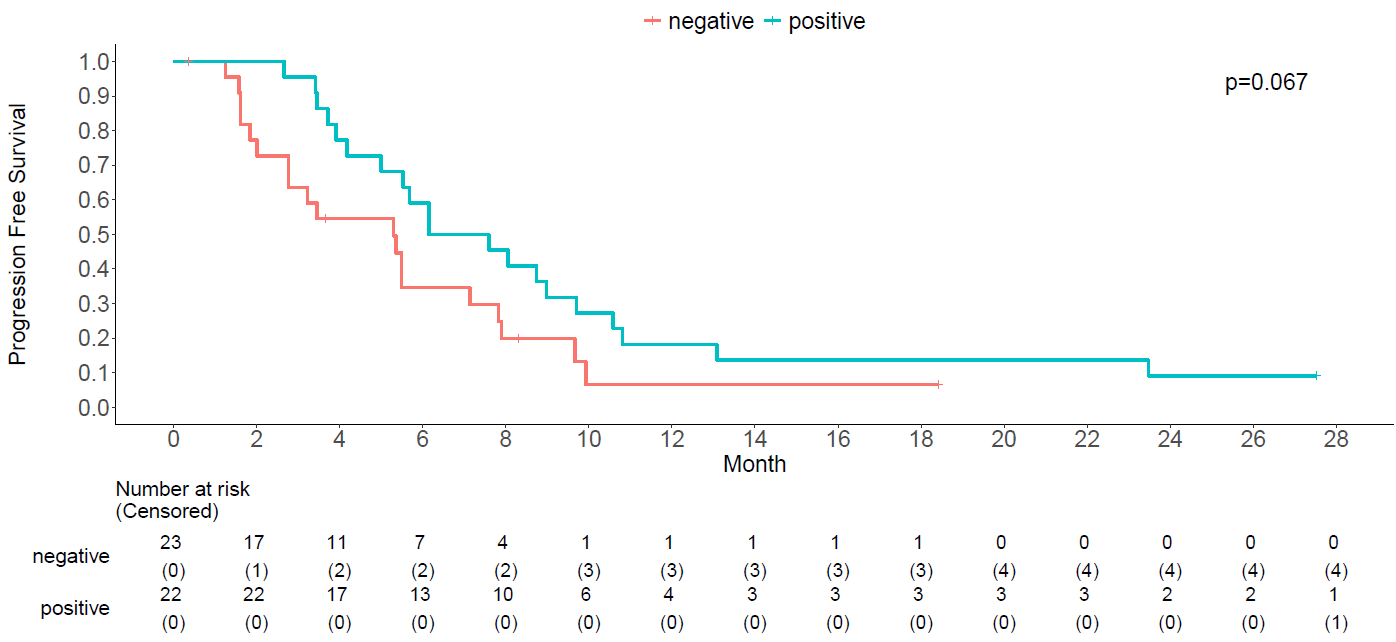


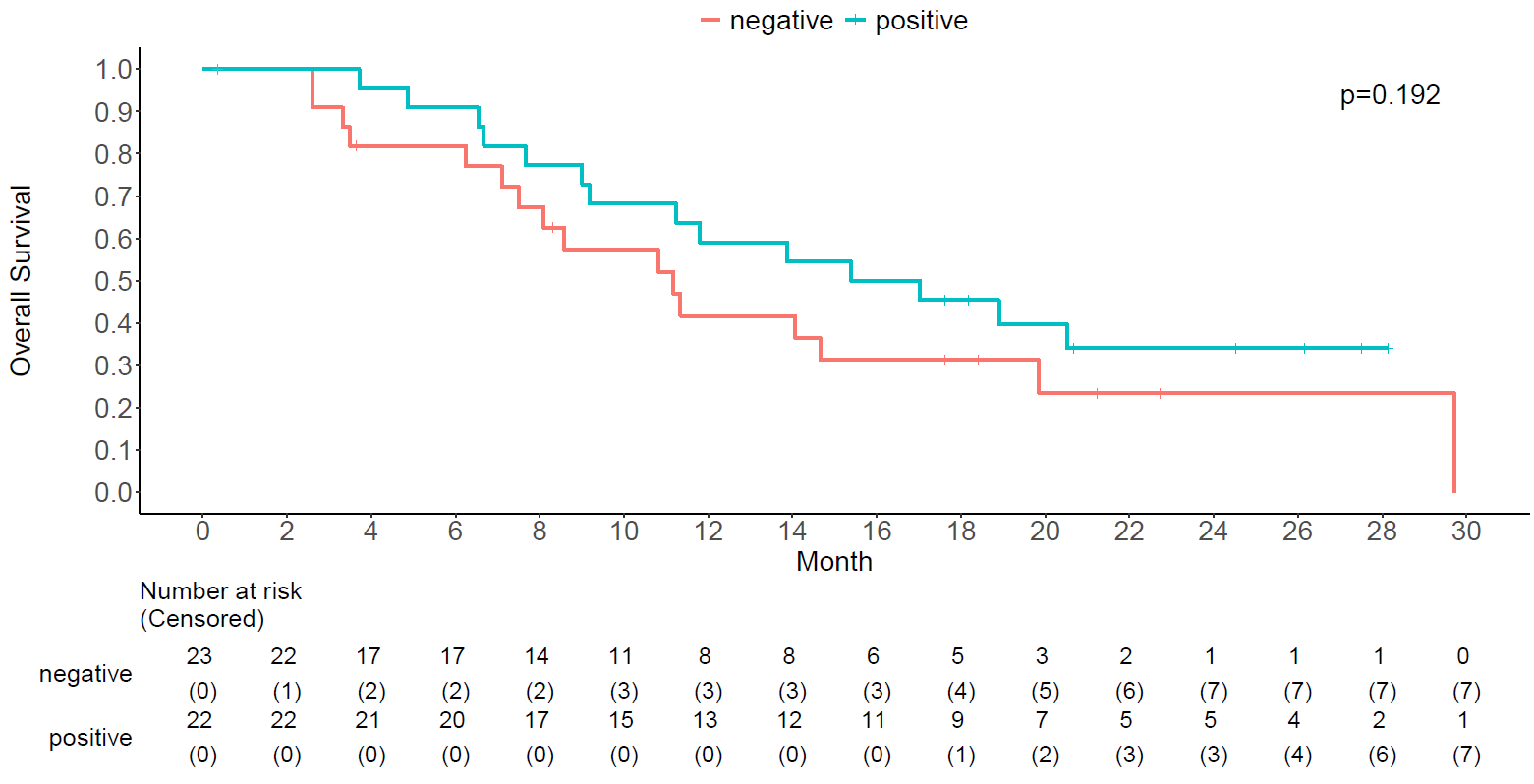


c.


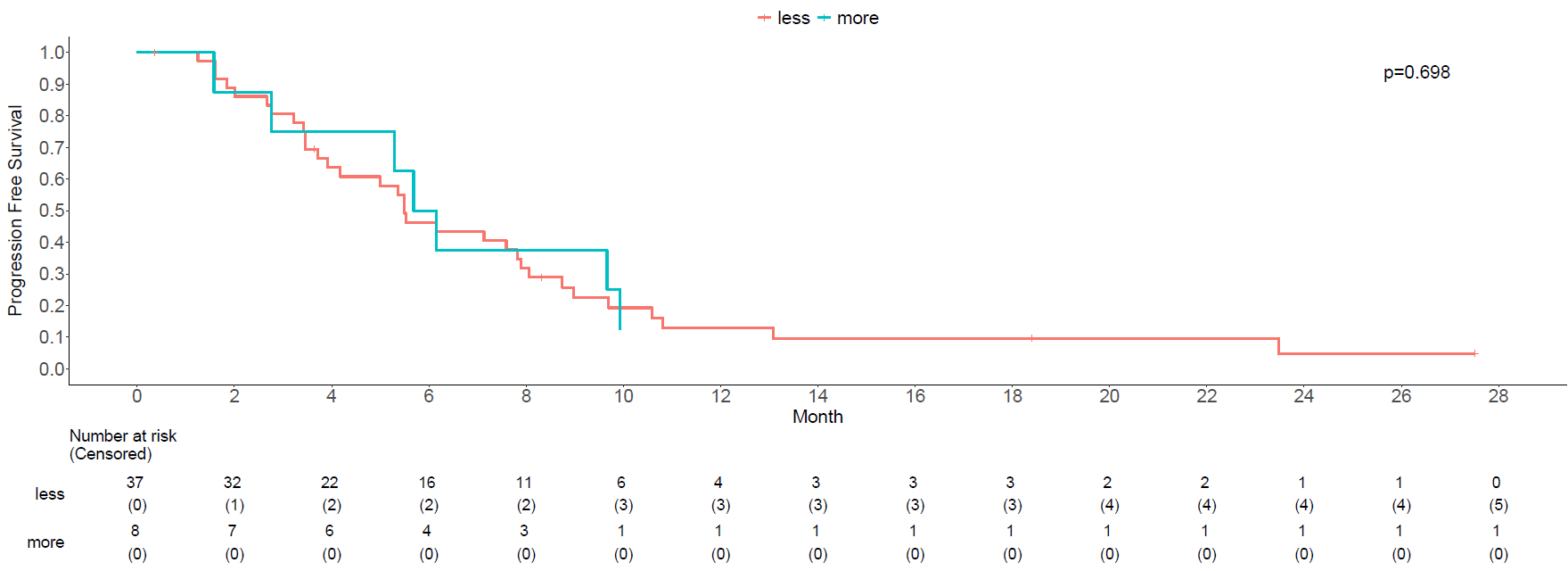


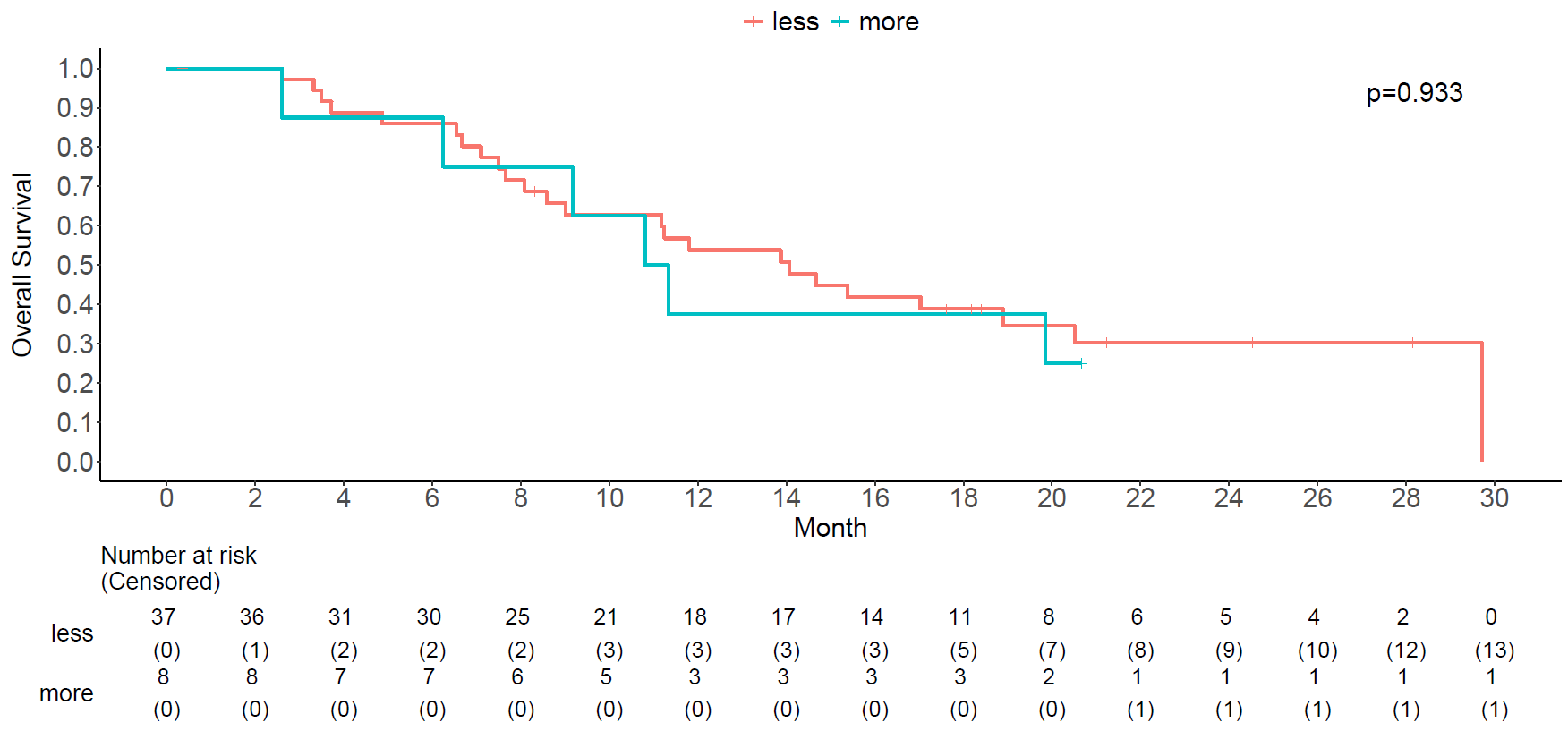

Supplement: Supplementary file 1 — Appendix S1: cam471533‐sup‐0001‐AppendixS1.docx. [file CAM4-15-e71533-s001.docx]
